# IndiSPENsable for X Chromosome Inactivation and Gene Silencing

**DOI:** 10.3390/epigenomes7040028

**Published:** 2023-11-02

**Authors:** Corinne Kaufmann, Anton Wutz

**Affiliations:** Institute of Molecular Health Sciences, Department of Biology, Swiss Federal Institute of Technology ETH Hönggerberg, 8093 Zurich, Switzerland; corinne.kaufmann@biol.ethz.ch

**Keywords:** SPEN, X chromosome inactivation, RRM, SPOC domain, intrinsic disorder, gene regulation, dosage compensation

## Abstract

For about 30 years, SPEN has been the subject of research in many different fields due to its variety of functions and its conservation throughout a wide spectrum of species, like worms, arthropods, and vertebrates. To date, 216 orthologues have been documented. SPEN had been studied for its role in gene regulation in the context of cell signaling, including the NOTCH or nuclear hormone receptor signaling pathways. More recently, SPEN has been identified as a major regulator of initiation of chromosome-wide gene silencing during X chromosome inactivation (XCI) in mammals, where its function remains to be fully understood. Dependent on the biological context, SPEN functions via mechanisms which include different domains. While some domains of SPEN are highly conserved in sequence and secondary structure, species-to-species differences exist that might lead to mechanistic differences. Initiation of XCI appears to be different between humans and mice, which raises additional questions about the extent of generalization of SPEN’s function in XCI. In this review, we dissect the mechanism of SPEN in XCI. We discuss its subregions and domains, focusing on its role as a major regulator. We further highlight species-related research, specifically of mouse and human SPEN, with the aim to reveal and clarify potential species-to-species differences in SPEN’s function.

## 1. The Discovery of SPEN as a Transcriptional Regulator

**Figure 1:**
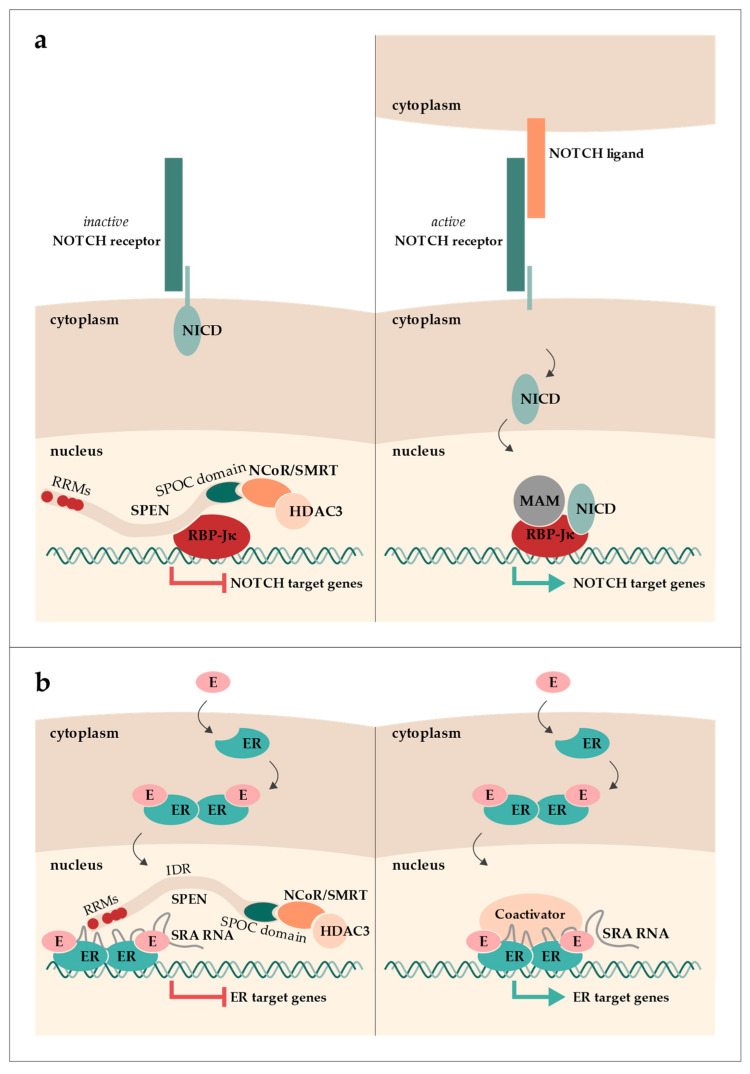
Graphical overview of SPEN’s roles in the NOTCH (**a**) and estrogen (**b**) signaling pathways. (**a**) In the absence of NOTCH signaling, SPEN binds to the transcription factor RBP-Jκ and recruits NCoR/SMRT bound to HDAC3, leading to the repression of NOTCH target gene expression. When the NOTCH receptor is activated, the NOTCH intracellular domain (NICD) is released into the nucleus and, together with mastermind (MAM), binds to RBP-Jκ, resulting in the expression of NOTCH target genes. (**b**) Estrogen (E) binding to the estrogen receptor (ER) induces a conformational change. ER dimerizes and translocates into the nucleus, where it can interact with either co-activators or co-repressors. SRA RNA can interact with both co-activators or co-repressors. When SPEN is bound to SRA and the ER dimer, ER target gene expression is repressed.

SPEN (aliases: SHARP and MINT; hereafter, the protein is referred to as SPEN, with a preference for the human nomenclature, also applicable to other genes/proteins, when referring to both mouse and human) was first described in *Drosophila melanogaster* as Split ends. Split ends was found to be involved in the development of axons in the peripheral neural system and received its name from a mutant phenotype that includes a distinctive pattern of veins in the wing margin, resembling the split ends of hair [1,2]. Further characterization predicted and finally characterized its N-terminal RNA-recognition motifs (RRMs) in a study screening for genes downstream of the Ras/MAPK/yan pathway in *Drosophila* [3,4]. Additionally, the C-terminal SPOC domain was discovered through sequence alignments of “SPEN-like” proteins found in worms, flies, and vertebrates, which are now considered orthologues of SPEN [5]. In mice, Spen was first identified in a far-Western blot analysis as an interactor of homeodomain transcription repressor Msx2 (its alias, Mint, stands for **M**sx2-**i**ntracting **n**uclear **t**arget protein) [6]. Msx1 and Msx2 are transcriptional repressors with an important role in stage-specific gene expression in osteoblast development of the skull [6]. The first reports of SPEN in humans revealed its function as a transcriptional regulator by associating with the nuclear receptor co-repressor (NCoR; alias NcoR1) and silencing mediator of retinoic acid and thyroid hormone receptor (SMRT; alias NcoR2) (its alias, SHARP, stands for **S**MRT/**H**DAC1-**a**ssociated **r**epressor **p**rotein) [7]. NCoR and SMRT are co-repressors of numerous transcription factors, which recruit and directly interact with histone deacetylases (HDACs). Both NCoR and SMRT form a strong complex with HDAC3. This complex formation is required for HDAC3 activity and thus histone deacetylation [8,9]. Around the same time, human SPEN was demonstrated to interact with a transcription factor of the NOTCH signaling pathway, namely RBP-Jκ (recombination signal binding protein for immunoglobulin kappa J region) [10]. The NOTCH signaling pathway is a highly conserved pathway important in cell fate decisions and lineage identity as well as cell proliferation and apoptosis. RPB-Jκ interacts with SPEN in the absence of NOTCH signaling and represses transcription by recruiting NCoR/SMRT via SPEN (Figure 1a). Upon NOTCH receptor activation, RBP-Jκ complexes with the NOTCH intracellular domain (NICD) and mastermind (MAM). This complex then activates the expression of NOTCH target genes (reviewed in [11]).

Another interaction of human SPEN was demonstrated in vitro, namely with the long non-coding RNA (lncRNA) steroid receptor RNA activator (SRA) [7]. While SPEN was shown to be a nuclear receptor (NR) co-repressor, SRA acts as an NR co-activator, particularly for the estrogen receptor (ER) [12,13,14]. By binding SRA, SPEN represses SRA’s NR-activating function (Figure 1b) [7].

In 2015, Spen was identified as a crucial factor in X chromosome inactivation (XCI) in mice, bridging the long non-coding RNA (lncRNA) Xist to downstream gene repressive processes and leading to a stably inactivated X chromosome [15,16,17,18,19]. XCI takes place in female mammalian cells to regulate the doubled gene dosage from the two X chromosomes compared to the single X chromosome in male cells [20]. During XCI, more than 400 genes are subject to transcriptional silencing [21]. XCI is mediated by the *cis*-acting Xist RNA, which is expressed and subsequently spreads along the future silenced X chromosome (future Xi) [22]. When expressed, Xist RNA initially establishes a repressive nuclear compartment at the non-genic chromatin regions of the X chromosome in mice [23]. Upon deacetylation of histone H3 and H4 by histone deacetylase 3 (HDAC3), Polycomb repressive complexes (PRCs) are recruited to those sites. PRCs lead to the deposition of histone modifications, specifically H2AK119ub (by PRC1) and H3K27me3 (by PRC2), providing a platform on which genes can become repressed [24] (reviewed in [25]).

## 2. XIST RNA and X Chromosome Inactivation in Humans and Mice

XIST RNA consists of several repeat elements (A-F repeats), each of which is involved in specific interactions or functions. The conserved A-repeat (RepA) was shown to be required for gene silencing in XCI in mammals [26]. However, independently of gene silencing, RepA-deleted Xist RNA (*Xist*ΔRepA) can still coat the future Xi, and the repressive compartment can still form in mouse embryonic stem cells (ESCs) [26,27]. Also, RNA polymerase II (RNA PolII), which is associated with active gene expression, was shown to be excluded from the compartment in *Xist*ΔRepA ESCs to an equal degree as in wild-type ESCs [23].

**Figure 2:**
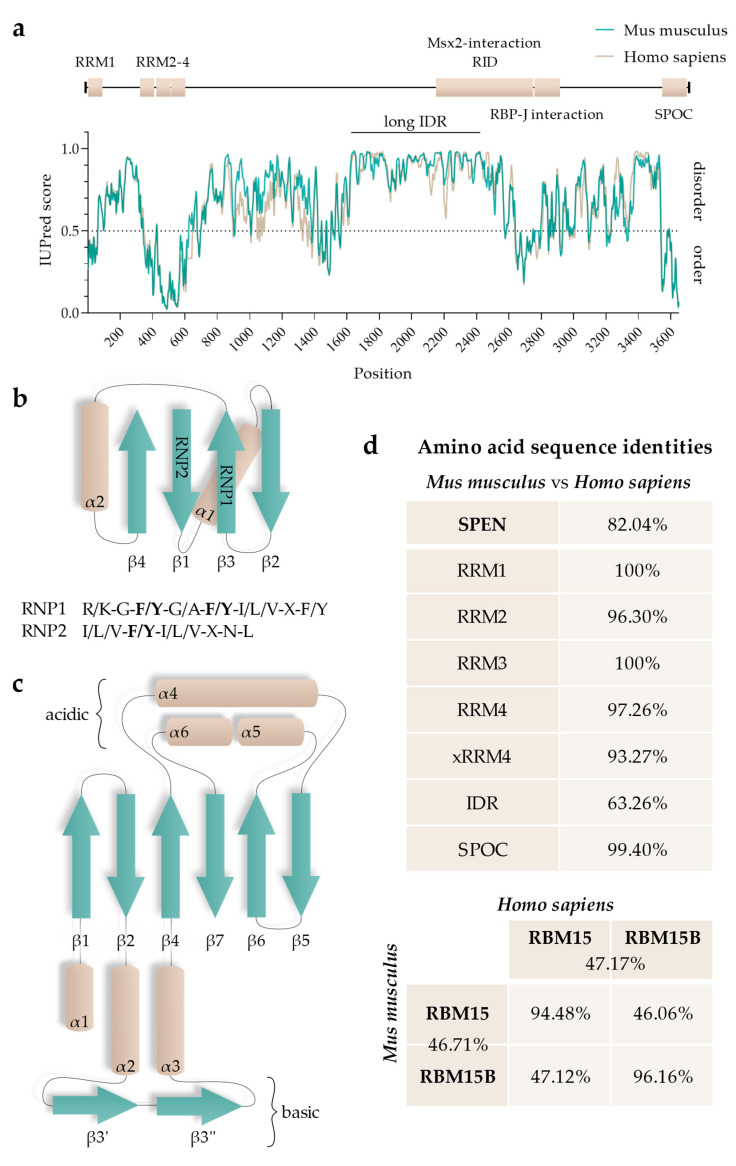
Functional regions of SPEN: (**a**) Graphical overview of SPEN’s functional domains in respect to the disorder prediction of human (Q96T58) and mouse (Q62504) SPEN using IUPred3 (long) (https://iupred3.elte.hu/plot, accessed on 15 September 2023). Values > 0.5 predict disordered and values < 0.5 predict ordered residues. The longest continuous IDR is highlighted (“long IDR”). (**b**) Secondary structure topology of RRMs. Aromatic residues of RNP1 and RNP2 are highlighted (bold) in their consensus sequences. (**c**) Secondary structure topology of the SPOC domain. (**d**) Amino acid identity measures obtained from identity matrices of ClustalW alignments of human SPEN (Q96T58), RBM15 (Q96T37), and RBM15B (Q8NT2) and mouse Spen (Q62504), Rbm15 (Q0VBL3), and Rbm15b (Q6PHZ5) using CLUSTAL O (1.2.4) (https://www.ebi.ac.uk/tools/msa/clustalo, accessed on 17 September 2023).

RepA has multiple interactors, such as SPEN, RNA-binding protein 15 and 15B (RBM15 and RBM15B, respectively), WT1 Associated Protein (WTAP), and Lamin B receptor (LBR) [16,19,26,28,29,30]. RBM15 and RBM15B are RNA-interacting proteins that guide the RNA methyltransferase complex WMM (WTAP/METTL3 (methyltransferase like 3)/METTL14 (methyltransferase like 14)), thereby regulating N6-methyladenosine modification (m6A) of different RNAs, including Xist RNA [29]. RBM15 and RBM15B are members of the small SPEN-like protein family (SSLP; as defined by [31]). Both RBM15 and RBM15B exhibit a very similar domain topology as SPEN but are only ~30% of SPEN’s size. Even though they display domain similarities, RBM15 and RBM15B have very little sequence identity with SPEN. Expectedly, they also differ in function. Furthermore, RBM15 and RBM15B share only 46.71% and 47.17% of amino acid sequence identity between mice and humans, respectively (Figure 2).

The direct interaction of Xist RNA B- and C-repeat with hnRNPK was shown to be required for the recruitment of PRC1 and PRC2 [32,33,34]. Contrary to mouse Xist RNA, the human XIST RNA E-repeat was shown to be additionally required for the recruitment of PRC2 and H3K27me3 enrichment [35] (Xist RNA regions and function reviewed in [36]).

In the mouse embryo, XCI was demonstrated to occur as early as in the 2-cell stage during pre-implantation development. At this stage, *Xist* is exclusively expressed from the paternal allele, leading to the transcriptional silencing of the paternal X chromosome (imprinted XCI) [37,38]. The paternal X chromosome remains silenced in the extraembryonic tissues. However, cells of the inner cell mass (ICM), which give rise to the embryo proper, reactivate it [37,38,39]. In cells with reactivated X chromosomes, another wave of XCI occurs in the post-implantation epiblast between embryonic day (E) 4.5 and E6.5. Unlike in imprinted XCI, both X chromosomes now have an equal chance of being inactivated, independent of the parental origin (random XCI) [38,40].

In the human embryo, *XIST* expression can be detected around the 4- to 8-cell stage [41,42], indicating a similar timing and mechanism as in the mouse embryo. However, XCI in human embryonic tissue was found to be random [43,44]. Interestingly, in the human blastocyst embryos, XIST RNA clusters often occur on both X chromosomes. This was also observed for cells in the ICM, indicating that both X chromosomes remain in an active state (XaXa) despite XIST RNA coating [42,45,46,47]. Furthermore, *XIST* upregulation is observed in both male and female embryos. The onset of chromosome-wide XCI occurs at the blastocyst stage prior to implantation (between E4 and E5) [45,46].

Mouse ESCs are readily available and constitute a suitable model for investigating XCI since they reflect the cell state of the pre-implantation embryo (XaXa). Human ESCs are derived from in vitro fertilized oocytes that were cultured in the blastocyst stage [48,49,50]. They are usually cultured in the primed state (XaXi), whereas there is variability in the cell lines’ XCI states and also instability of *XIST* expression in prolonged cultures [51,52] (reviewed in [53]). Due to different genetic backgrounds and a large gene pool, human ESC lines are rather variable. Together with the more sensitive nature of human ESCs in culture, cell lines can be heterogenous [54]. Recent efforts in stem cell research have allowed the resetting of the cells to their naïve state [55,56,57,58,59]. Transcriptomics have revealed that naïve human ESCs resemble the pre-implantation epiblast and have bi-allelic X chromosomal expression (XaXa). However, very few cells contain two XIST RNA clusters on both of their X chromosomes, unlike ICM cells from the human embryo [42,58,60,61,62]. XCI state variabilities and genomic instabilities are also observed in naïve human ESCs (reviewed in [53]). Naïve human ESCs typically have only one XIST RNA-coated X chromosome, but unlike in mouse cells, both in vitro and in vivo observations show that XIST RNA is not immediately followed by gene repression in human cells [42,60].

## 3. Function of SPEN in Mouse X Chromosome Inactivation

In mice, expression of *Xist* activates a cascade of repressive processes leading to the deposition of inactivating chromatin modifications that enable a stably inactivated state of the X chromosome (reviewed in [63]).

Xist RNA coating of the future inactive X chromosome (Xi) leads to the recruitment of Spen to the Xi. Spen further interacts with NCoR/SMRT, which bind and activate Hdac3, ultimately resulting in the removal of active histone marks [7,8,9,30,64]. In vitro studies have shown that Spen is required for the initial expression of *Xist* by repressing *Tsix* expression. Tsix is an X-chromosomally expressed lncRNA that acts as an antisense transcript of *Xist* and genetically fully overlaps with it. Tsix RNA was demonstrated to inhibit the expression of *Xist* in mouse ESCs [65,66,67,68,69]. These findings would suggest a function of Spen in the initiation phase of XCI by negatively regulating *Tsix* expression and thus allowing *Xist* upregulation. Another study showed that during XCI maintenance, depleting Spen resulted in an upregulation of *Xist* expression. This was observed in both differentiated mouse ESCs and in ESCs with induced *Xist* expression [70]. Interestingly, elevated Xist RNA levels caused spreading of Xist RNA to adjacent chromosomes. Spen’s negative regulation of *Xist* expression in differentiated or *Xist*-induced cells further suggests a function for Spen in ensuring Xist RNA spreading is restricted to the X chromosome during the establishment of XCI. Furthermore, depletion of Spen in fully differentiated mouse neuronal progenitor cells (NPCs) did not lead to the reactivation of silenced genes [30]. These findings suggest that Spen negatively regulates *Xist* expression during XCI maintenance but is not required for gene silencing at this stage.

The requirement of Spen for XCI in mice is underlined by findings obtained by in vivo studies. First knockout studies showed that homozygous *Spen* mutant embryos have a morphologically abnormal pancreas and heart and are not viable [71]. Lethality starts around embryonic day (E) 12.5, and at around E16.5, no homozygous *Spen* mutant embryos could be obtained. While this study did not differentiate between male and female embryos, the Mendelian segregation of the genotypes indicate that sexes are not affected differently by the *Spen* mutation. In this study, the *Spen* mutant allele was generated by insertion of a PGK-Neo cassette for disrupting gene expression in exon 12 [71]. Truncated Spen mRNA transcripts were detected at E12.5 [71]. Consequently, the expression of the remaining coding region might result in a truncated Spen protein containing the RRMs but lacking the Rbp-Jκ interaction region and the SPOC domain. In contrast to this study, a paternally transmitted *Xist* knockout was shown to lead to female-specific lethality around E8.5 due to defective imprinted XCI [72]. Hence, the lethality of *Spen* mutants appears remarkably late considering its requirement in both imprinted and random XCI in mouse ESCs. The late lethality could be explained if Spen was not required for imprinted but solely for random XCI in the epiblast. A subsequent study, however, revealed that mouse embryos with *Spen* knockout had severely impacted imprinted XCI [30]. Knockouts were generated by the backcrossing of female mice with conditional oocyte-specific *Spen* deletion to *Spen*^+/−^ male mice. Interestingly, in embryos with only the oocyte-specific deletion, imprinted XCI was unaffected [30]. These observations suggest that Spen is indeed required for imprinted XCI, but zygotic expression of *Spen* is sufficient for this role. In this study, mutant mice were evaluated at E3.5. Therefore, no information about timing of lethality caused by the *Spen* knockout was obtained.

The knockout or mutation of *Spen* likely affects mice beyond XCI. Other pathways with involvement of Spen may be impacted as well, namely the (1) Sra, (2) Msx2, (3) NCoR/Smrt, and (4) Notch pathways. To understand the late lethality in *Spen* knockout mice, it may be helpful to compare these observations to other mouse knockout studies related to the other pathways. (1) Interestingly, *Sra1* (encodes the lncRNA Sra and additionally the Sra protein (SRAP)) knockout mice are viable, and genotyping revealed the expected Mendelian ratio, suggesting no sex-related effects of the mutation [73]. *Sra1*^+/−^ and *Sra1*^−/−^ mice appeared normal, and *Sra1*^−/−^ mice were shown to be resistant to high-fat-diet-induced obesity [73]. (2) *Msx2*^+/−^ mice are unaffected, while *Msx2*^−/−^ mice are viable but were found to have a defect in calvarial development [74]. (3) *Ncor*^−/−^ mice were also embryonic lethal, where the majority were found to have died by E15.5 due to neurologic and hematologic defects [75]. *Smrt*^−/−^ mice were found to be embryonic lethal due to impaired cardiac ventricular development [76]. The phenotype was detectable around E11.5, and by E16.5, the majority of the embryos had died [76]. *Hdac*^+/−^ mice are viable, while *Hdac*^−/−^ mice were found to be embryonic lethal around E8.5-E9.5 due to defects in gastrulation [77,78]. (4) There are four Notch receptors encoded by *Notch1*, *Notch2*, *Notch3*, and *Notch4*. *Notch1*^+/−^ mice were viable, while *Notch1*^−/−^ embryos did not survive past E9.5 [79,80,81]. *Notch2*^−/−^ mutations led to embryonic lethality around E11.5 [82]. On the other hand, both *Notch3*^−/−^ and *Notch4*^−/−^ mice were found to be viable [81,83].

Among these observations, *Ncor*^−/−^, *Smrt*^−/−^, and *Notch2*^−/−^ mice exhibited a timing of lethality similar to *Spen* mutant embryos. The Notch signaling could indeed be involved in the phenotype of homozygous *Spen* mutant embryos from Kuroda et al. (2003) [71]. Abnormalities in these mutant embryos affect the pancreas and heart, organs whose embryogenesis is regulated by Notch signaling. Additionally, even if *Spen* truncation were to result in a truncated but functional protein, it lacks the Rbp-Jκ binding site. This would further indicate that in these *Spen* mutant embryos, Notch signaling is likely affected.

## 4. Function of SPEN in Human X Chromosome Inactivation

While most studies of SPEN are performed in mice or mouse cells, SPEN’s function in human XCI is less clear. In mouse ESCs, Xist RNA coating leads to immediate gene repression by recruiting Spen. This recruitment is thought to establish an inactivated X chromosome. However, in human cells, this early step in XCI differs. Human female pre-implantation embryos possess two XIST RNA clusters without indication of X chromosome silencing. Enrichment of H3K27me3 was observed on the X chromosomes in naïve human ESCs (XaXa) but was not sufficient to induce silencing [60]. Indeed, in naïve human ESCs, only subtle repression or dampening of X chromosomal gene expression was observed in cells with bi-allelic *Xist* expression [46,60]. This observation indicates that in early human embryonic cells, XIST RNA does not engage silencing pathways to the same extent as in mice during the initiation of XCI. This lack of immediate and definite consequences of XIST RNA coating, as seen in mice, is surprising, and the origin and extent of the differences in XCI mechanisms between humans and mice are not well understood.

A difference between human and mouse XCI is the lncRNA TSIX and the lncRNA XACT. Human TSIX RNA is shorter and does not fully overlap with *Xist* compared to mouse Tsix RNA. Human TSIX RNA was shown to be co-expressed with *XIST* from the Xi and seems unable to repress *XIST* expression [84]. The human-exclusive XACT RNA, however, was shown to impact XIST RNA localization and its silencing function. XACT and XIST RNA were demonstrated to co-accumulate on the active X chromosomes in the XaXa but also XaXi state in human pre-implantation embryos and in naïve ESCs [42]. Notably, XACT and XIST RNA localization barely overlapped on the X chromosomes, and XIST RNA accumulation was rather dispersed [42]. By transgenically introducing *XACT* into one X chromosome of mouse ESCs and inducing endogenous *Xist* expression, it could be demonstrated that *Xist* was preferentially expressed from the X chromosome without the *XACT* transgene [42]. These findings suggest an antagonistic mechanism of XIST and XACT RNA and indicate that *XACT* expression or its RNA has a regulatory effect on XIST RNA accumulation and silencing. However, the mechanism of XACT RNA in preventing gene repression remains to be understood.

The extent of SPEN’s involvement in early human XCI is difficult to assess due to understandable ethical concerns regarding early human embryo research. Single-cell RNA-sequencing analysis of human and mouse pre-implantation embryos reveals that SPEN mRNA levels are rather low but detectable from the stage of the zygote to late ICM in both human and mouse embryos (range 1.1 to 6.7 FPKM) [85]. Furthermore, SPEN expression is in similar ranges when comparing the same developmental stages between humans and mice, whereas there is slightly more mRNA detected in late human ICM when compared to Spen mRNA levels in late mouse ICM (6.7 and 2.7 FPKM, respectively). These findings indicate that in human pre-implantation embryos, SPEN has the same if not slightly higher abundance compared to mouse embryos.

Clinical research involving SPEN in humans primarily focuses on the NOTCH signaling pathway, which results in limited patient data regarding SPEN’s role in human XCI. In a recent study, it was shown that truncating mutations of *SPEN* cause neurodevelopmental disorders and intellectual disabilities in a haploinsufficient manner [86]. Truncating mutations of *SPEN* were found distributed throughout the entire gene in various patients. Interestingly, truncating variants at the C-terminus did have the same effect and thus resulted in the same severity of phenotype as truncations closer to the N-terminus. This observation suggests that the SPOC domain is needed for SPEN’s function in humans. Data further revealed that haploinsufficiency of SPEN resulted in differential DNA methylation of the X chromosome in females. While global DNA methylation in the cell was not significantly affected, DNA methylation occurred in a distinctive epi-signature on the X chromosome [86]. These observations might indicate a role for SPEN in human XCI.

## 5. Functional Regions of SPEN

In the following paragraphs, we discuss SPEN’s domains and regions in relation to its function in XCI and examine their conservation between humans and mice.

### 5.1. RNA-Recognition Motifs

SPEN proteins possess RNA-binding domains within an N-terminal position. The RNA-recognition motif (RRM) is defined as a ~90-amino-acid-long eukaryotic protein region that is able to bind single-stranded RNA (ssRNA) [87]. The topology of the secondary structure is highly conserved and consists of three β-sheets and two α-helices, where the adjacent β-sheets form a surface (Figure 2) [88]. Structured parts of the RRMs contain highly conserved amino acid sequences. β3 and β1 contain two conserved consensus sequences, called RNP1 and RNP2, respectively. Notably, the amino acid sequence of RNP2 is less conserved than that of RNP1 [89,90].

**Figure 3:**
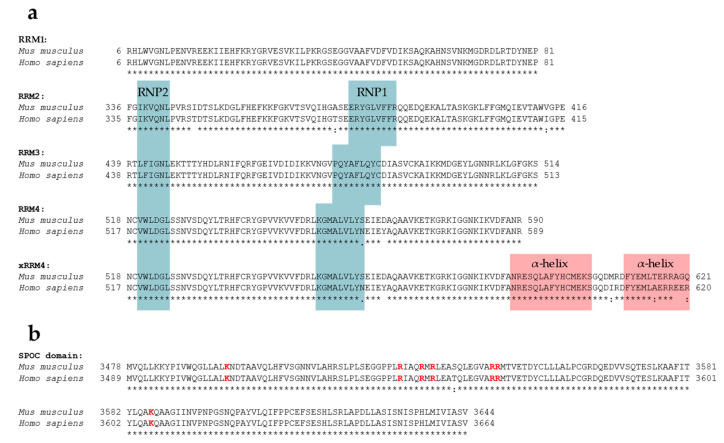
ClustalW alignment of SPEN’s functional regions RRM2-4 and SPOC domain of human and mouse SPEN. The symbol ‘*’ below the alignments indicates an identical residue, while ‘:’ and ‘.’ indicate a conserved or semi-conserved substitution, respectively. No symbol indicates a mismatch or an alignment gap. (**a**) Alignment of human (Q96T58) and mouse (Q62504)RRM1, RRM2, RRM3, RRM4, and RRM4 including the extending α-helix (xRRM4). RNP1 and RNP2 consensus sequences obtained from [96] are highlighted in blue (note that for RRM1, RNP1 and RNP2 have not been annotated). Extending α-helices of xRRM4 are highlighted in red. (**b**) Alignment of human (Q96T58) and mouse (Q62504) SPOC domain. Conserved basic patch residues are highlighted in red. Alignments were obtained using CLUSTAL O (1.2.4) (https://www.ebi.ac.uk/tools/msa/clustalo, accessed on 17 September 2023).

SPEN’s RRMs are highly conserved; RRM1, RRM2, RRM3, and RRM4 of humans and mice share 100%, 96.30%, 100%, and 97.26% amino acid sequence identity, respectively (Figure 2, see Figure 3 for ClustalW alignment). Arieti et al. (2014) obtained crystal structures of human SPEN RRM2-4 [91]. Notably, RRM1 was not included in this structural analysis. The crystal structures reveal that RRM2 is flexibly linked to RRM3 and RRM4 with an α-helix-forming linker of 20 amino acids [91]. RRM3 and RRM4, on the other hand, are linked by seven highly conserved amino acids in a much less flexible manner, arranging RRM3 and RRM4 into a platform. The β-sheet surface that acts as an ssRNA binding site of the RRM3 is accessible. The β-sheet surface of RRM4, however, interacts with the first of two C-terminal alpha helices of RRM4 (human: residues 588–602 and 609–620; mouse analogs: 589–603 and 610–621) [91]. Such atypical RRMs with structural extensions were described and defined as xRRM by Singh et al. in 2012 [92]. In that study, an xRRM was identified in *Tetrahymena* p65, a member of the LARP7 protein family with two RRMs. The crystal structure of the free protein revealed a short extending helix of RRM2 with an unstructured C-terminal part. In the complex with the interacting telomerase RNA (TER), the extending helix formed a longer and slightly bent helix, allowing it to bind dsRNA regions of TER. This elongation was shown to be essential for p65–TER interaction. Notably, the extension of the α-helix enables binding to double-stranded RNA (dsRNA) in addition to the ssRNA interaction that occurs at the β-sheet surface of RRMs [92]. Additional xRRMs have been identified in other RNA-binding proteins, such as the human U1A protein, which binds the U1 RNA hairpin ii. Upon RNA binding, the extending α-helix undergoes a change in orientation, which is required for protein–RNA interaction. The interaction ultimately stabilizes the new orientation of the α-helix [93]. The extending α-helix of SPEN xRRM4 showed a conserved secondary structure placement compared to xRRMs of p65 and hnRNP F, which suggests that SPEN’s xRRM4 could have a similar regulatory function in RNA interaction [91]. To investigate this potential regulatory function of SPEN’s xRRM4, Arieti et al. (2014) crystallized different RRM mutants in complex with the hairpin substructure H12-H13 of the lncRNA SRA [91]. While RRM2 deletion did not affect binding affinity, deletion of RRM4 along with the extending α-helix impaired the binding of H12-H13 [91]. The dispensability of RRM2 is in agreement with its lower conservation among SPEN orthologues [4]. Mutations in RRM3 (RRM3mut) led to an instable binding of H12-H13 [91]. Interestingly, amino acid changes in mouse RRM3 (mRRM3mut), corresponding to human RRM3mut studied by Arieti et al. (2014), resulted in a binding affinity to Xist RNA RepA reduced by half. This was demonstrated by irCLIP-seq (UV-C crosslinking and immunoprecipitation method) in a later study [91,94]. This observation suggests that RRM3 follows a similar mechanism in SRA RNA and Xist RNA binding. However, whether mRRM3mut has the same impact on the RNA interaction of Spen with Xist compared to SRA RNA is difficult to estimate due to the different experimental approaches and different model species. In contrast to SPEN, RBM15 and RBM15B are not exclusively recruited to repeat A of Xist RNA [29]. Nonetheless, a recent study specified the co-dependency of RBM15 RRMs in XIST RNA binding in human cells and showed that RRM2-3 (analogs to SPEN RRM3-4) could bind XIST RNA RepA with a higher affinity than RRM1-2 (analogs to SPEN2-3), which is consistent with the interactions of SPEN RRMs with Xist or SRA RNA [91,95].

Competition experiments with other RNA sequences showed that secondary structures, for example, the stem-loop structure of H12–H13, were required for the interaction of SPEN RRMs with RNA, indicating binding specificity [91]. For the binding of XIST RepA by SPEN RRMs in mice and humans, SPEN was demonstrated to bind specific structural features at AU-rich regions where Xist/XIST RNA is single-stranded but adjacent to a double-stranded hairpin or within a small internal loop of the hairpin [15,19,94,97]. Interestingly, SPEN clusters when binding RepA in biochemical binding assays in vitro, which was not observed when SPEN was bound to other mRNAs [97]. *Xist* repeats are presumed to have originated from the insertion and duplication of transposable elements (TE), with RepA showing similarity to endogenous retroviruses (ERVs) [98]. A study has demonstrated that *Spen* knockout mouse ESCs exhibit a de-repression of a subset of endogenous retrovirus K (ERVK) TEs [94]. Additionally, irCLIP data revealed that Spen binds to ERV-derived RNA in the cell. These observations collectively suggest that Spen has a role in repressing specific viral TEs by interacting with their RNA [94]. Interestingly, Spen was found to bind to mutant Xist RNA (*Xist*-ERV), in which the RepA of a doxycycline-inducible *Xist* was replaced by an ERV sequence using CRISPR-Cas9 genome editing. Notably, the expression of *Xist*-ERV was only inducible in the presence of HDAC1/2 inhibition. Knockdown experiments indicated that Spen is likely recruited by *Xist*-ERV, resulting in the local silencing of the transgenic *Xist*-ERV [94]. Altogether, studies investigating the interaction of XIST RepA with SPEN suggest that both the secondary structure of the RNA and specific affinities to various consensus sequences contribute to SPEN’s specificity for XIST RepA.

XIST RNA RepA can be bound not only by SPEN’s RRMs but also by the RRMs of RBM15/RBM15B in humans and mice. RBM15/RBM15B binding might contribute to m6A deposition on XIST RNA by interactions with WTAP/METTL3. WTAP, which has also been demonstrated to bind Xist RNA, has been suggested to require Xist RepA for the interaction [15]. This requirement suggests a link between gene repression and m6A RNA methylation. However, the contributions of single m6A sites to the function of XIST RNA are not clear. A study by Nesterova et al. (2019) conducted in mouse ESCs revealed that the deletion of specific regions of a major m6A peak downstream of RepA results in either no or only a slight deficit in X chromosome silencing [99]. These findings suggest that m6A sites of Xist RNA potentially interrelate in a hierarchical manner [99]. *Mettl3*, *Wtap*, or *Rbm15* knockouts did not impact X chromosomal gene silencing in this study [99]. Another study, however, reported that the global reduction of Xist RNA m6A modifications, achieved by Ythdc1 (YTH domain containing 1; an m6A reader), Mettl3, or combined Rbm15 and Rbm15b knockdown, prevented Xist-mediated X chromosome silencing in mouse ESCs [29]. The findings of those two studies suggest a redundancy of Rbm15 and Rbm15B in respect to their function in XCI. However, it remains unclear if global m6A depletion of Xist RNA can abrogate X chromosomal gene silencing or if systemic m6A reduction affects other pathways that indirectly impact XCI.

Interestingly, Ythdc1 was found to bind Xist RNA m6A and enrich the Xist RNA compartment in the nucleus [29]. In the study of Patil et al. (2016), the impact on X chromosome silencing due to Ythdc1 knockdown could be rescued by artificially tethering human YTHDC1 to Xist RNA in mouse ESCs [29]. YTHDC1 interaction with Xist RNA is not well understood. Interestingly, next to RBM15, PRC1, and PRC2, SPEN was identified as an interaction partner of YTHDC1 via its SPOC domain in mouse ESCs and HEK293 cells [30,100]. The relationship between SPEN and YTHDC1 has not been unveiled yet, and the role of YTHDC1 in XCI remains to be fully explored.

### 5.2. SPOC Domain

The C-terminal SPOC domain is highly conserved (57% identity between *D. melanogaster* Spen and *H. sapiens* SPEN) and has a unique sequence motif, which is found in SPEN proteins and short SPEN-like proteins (SSLPs; <800 amino acids) [31]. Examples for SSLPs are Spenito (*D. melanogaster*) and its orthologue RBM15 in both mice and humans.

The SPOC domains of mouse and human SPEN share 99.40% amino acid identity (Figure 2, see Figure 3 for ClustalW alignment). The high degree of sequence conservation correspondingly results in a conserved secondary structure. SPOC domains comprise a β-barrel structure with seven β-strands (β1–β7) surrounded by six α-helices (α1–α6) (Figure 2) [101]. A potential region for protein–protein interactions is a non-polar groove surrounded by two proline-rich loops. The groove is between β3′ (a disrupted β3-strand results in β3 and β3′) and β5-strand. Another groove potentially involved in protein–protein interactions can be found between the α1-helix, α6-helix, and the N-terminus of the α4-helix. Here, the cavity consists of hydrophobic amino acid residues and backbone carbonyl groups, resulting in a slightly acidic environment. While one side of the SPOC domain is negatively charged (α4-, α5-, and α6-helices on one side), the other side of the domain has a highly conserved basic patch (β3-strand, which is lysine- and arginine-rich) [101].

SPEN’s SPOC domain interacts with a number of factors. These include the phosphorylated C-terminal LSD motif (conserved between humans and mice) of NCoR/SMRT (NCoR: …YET**L***p***SD**SDD, SMRT: …YET**L***p***SD**SE) [7,64,102], the co-activator histone-lysine N-methyltransferase 2D (KMT2D; a H3K4 methyltransferase) [103], the CTD motif of RNA Polymerase II (RNA Pol II) phosphorylated at residue serine-5 (*p*S5) [30,102], and the m6A reader fragile X mental retardation 1 (FMR1) [102]. Structural analyses reveal that interactions of the SPEN SPOC domain with the LSD motif of NCoR/SMRT (*p*S2522) and the CTD of RNA pol II (*p*S5) is mediated by the basic patch residues K3516, R3552, and K3606 [102]. Additionally, residues R3552 and R3554 were shown to be necessary to bind an LSD peptide of NCoR/SMRT in an affinity resin assay (Figure 3) [7]. The conserved residue Y3602 of SPEN is involved in binding the second CTD repeat of the phosphorylated (*p*S5) RNA pol II, and R3548 is involved in electrostatic interactions with the negatively charged residues of the LSD region of its interaction partner NCoR/SMRT [102]. Another residue in the basic patch, R3566, causes electrostatic repulsion against the positively charged R507 of FMR1 and seems to reduce the binding affinity of FMR1 [102].

Upon initiation of XCI, SPEN is recruited to the Xi [15,19,69,97,102]. SPEN’s SPOC domain interacts with the NCoR/SMRT complex, which activates HDAC3. This leads to the subsequent removal of active histone marks on the future inactive X chromosome [7,9,30]. The interaction between NCoR/SMRT and HDAC3 takes place via the deacetylase-activation domain (DAD) of NCoR/SMRT [8,9]. HDAC3 was shown to be the only histone deacetylase crucial for histone deacetylation during the early stages of XCI [16,24]. Interestingly, the knockout of *Hdac3* in mouse ESCs leads to a delay but not abrogation of transcriptional silencing in XCI [24]. The *Spen* knockout, however, abolishes XCI and therefore has a more severe effect on XCI than the *Hdac3* knockout [30]. These observations indicate that there might be other factors recruited to the Xi by Spen, which act in addition and redundantly to Hdac3.

KMT2D was demonstrated to be another interaction partner of SPEN’s SPOC domain [103]. The interaction of KMT2D competes with NCoR/SMRT binding to the SPOC domain. Notably, the phosphorylation of the LSD motif of NCoR/SMRT enhances the binding affinity and shifts the interaction of the SPOC domain in favor of NCoR/SMRT [103].

Interestingly, Spen was shown to accumulate at promoters and enhancers but overlaps exclusively with Hdac3 at enhancers and with the nucleosome remodeling and deacetylation (NuRD) complex at promoters in mouse cells [30]. The NuRD complex also interacts with SPEN’s SPOC domain and is known for its regulation of the chromatin architecture during cell-state transitions, which leads to the deacetylation of H3K27 [104]. Therefore, SPEN might act via distinct pathways at enhancers and promoters to promote gene silencing by deacetylation of histones. However, to this day, little is known about the distinct silencing pathways of SPEN in XCI, and further studies are needed to investigate these pathways individually.

During the initiation phase of XCI, RNA PolII is excluded from the area of the Xist cluster [23]. Both Hdac3 or Spen knockdowns resulted in the loss of RNA PolII exclusion from Xist cluster areas in mouse ESCs when Xist expression was induced by doxycycline [16]. This observation suggests that both Hdac3 and Spen contribute to the exclusion of RNA PolII from the future inactive Xi. Even though the SPOC domain was demonstrated to directly interact with RNA PolII, deletion of the SPOC domain does not fully abolish this interaction. Nevertheless, deletion of the SPOC domain reduces nascent transcription at transcription start sites (TSSs) and gene bodies [102]. These findings imply that although the SPOC domain directly interacts with RNA PolII, other regions of SPEN are likely involved in mediating this interaction [102].

Both Hdac3 and Spen knockdown resulted in the loss of PRC2 recruitment to the X chromosome undergoing inactivation in mouse ESCs, as measured by the reduction of the PRC2 component EZH2 [16,19]. Earlier studies demonstrated that RepA-deleted Xist RNA strongly impacted PRC2 recruitment in early stages of ESC differentiation [26,27,105]. Although PRC2 recruitment at later stages of ESC differentiation is independent of RepA, these observations suggest a mechanism for the recruitment of PRC2 by Xist RNA RepA. This recruitment may depend on the presence or actions of Hdac3 and/or Spen at the Xi. However, the mechanism underlying PRC2 recruitment in XCI is not completely understood. Notably, no PRC2 components have been identified as direct SPOC domain interactors [30,103].

### 5.3. Intrinsically Disordered Regions

All SPEN-like proteins contain RNPs and a SPOC domain in a similar arrangement. SSLPs (small SPEN-like proteins, as described earlier) additionally contain glycine-arginine-rich (RGG) domains, which can destabilize RNA helices [31,106]. A striking difference in SPEN (in both humans and mice) compared to SSLPs lies in its remarkably long intrinsic disordered region (IDR), which likely contributes to functional differences between SPEN and other SPEN-like proteins (Figure 2).

IDRs are regions without a defined and stable 3D structure. They often contain disorder-promoting residues (such as Lys, Gln, Ser, Glu, and Pro), while they lack order-promoting residues (such as Cys, Trp, Ile, Tyr, Phe, Leu, His, Val, Asn, and Met) (reviewed in [107]). IDRs are often targets for post-translational modifications (PTMs), including phosphorylation [108] (reviewed in [109]). Furthermore, IDRs have been shown to be involved in protein–protein interactions, including the formation of concentration-dependent hetero- and/or homotypic assemblies [108,110]. Assembly formation has also been proposed to regulate liquid–liquid phase separations [111,112]. IDRs can transition between disordered and ordered structures and vice versa (called phenotypic switching), which can be caused by interactions and/or PTMs [109] (reviewed in [113]). In those ordered or disordered states, an IDR can have preferences towards one or several specific conformations (conformational or structural bias). As a result, IDRs are not considered “unstructured” but rather contain a number of possible conformations (reviewed in [114]). Needless to say, the longer an IDR is, the greater the number of possible conformations it can theoretically adopt [115].

Sequence composition of the IDR can be used to classify the IDR-containing proteins into “polar tracts” (enriched in polar residues and deficient in charged, hydrophobic, and proline residues), “polyelectrolytes” (either enriched in residues with positive or negative charges), and “polyampholytes” (equal amounts of positively and negatively charged residues). Depending on the class, an IDR has a different conformational bias, and therefore, certain conformations can be expected for each class (also called composition-to-conformation relationships) (reviewed in [116]). However, not only the sequence composition of an IDR but also the sequence pattern determines its conformation or conformational bias. Therefore, when predicting possible conformations, both the sequence composition and pattern have to be considered. Additionally, physicochemical properties of the IDR’s environment influence its conformation, including factors like temperature and the abundance of ligands or other proteins (reviewed in [114]). When the environment undergoes changes, the IDR’s intramolecular interactions can change and, subsequently, its conformation. These observations suggest that IDRs potentially act as physicochemical sensors (reviewed in [114]). The IDRs of SPEN have not been classified and characterized yet; thus, not much is known about its conformational or structural bias.

More than 30% of the human proteins contain IDRs (with a length of >30 residues), many of which are transcription factors and multifunctional enzymes [117,118]. The presence of IDRs is suggested to enable a phenotypic plasticity that contributes to the evolution of different cell types in eukaryotes (reviewed in [119]). SPEN is predicted to contain multiple IDRs, but there is one remarkably long IDR C-terminal to RRM4 spanning, with a few ordered gaps, to the SPOC domain. The longest continuous IDR is almost 1000 amino acids long, as predicted by IUPred2 and IUPred3 (Figure 2) [70]. Such long IDRs are unexpected and rare.

It was recently demonstrated that SPEN forms assemblies during its recruitment by XIST RNA to the Xi. SPEN’s accumulation occurs in a non-stoichiometric manner to XIST RNA and is dosage-dependent [70,120]. For the assembly formation, SPEN’s IDRs were shown to be required. Consequently, IDR deletion had a strong impact on XCI without losing the interaction between Spen and Xist RNA RepA in mouse ESCs [70,120]. Interestingly, the phenotype of IDR deletion could be rescued by fusing an IDR from a different protein to the N-terminal end of a transgenic rescue fragment of SpenΔIDR [70]. Rescue fragments could restore assembly formation and Spen’s localization to the Xi. Additionally, XCI could be rescued to a large extent, as evaluated by RNA FISH of X-chromosomal genes [70]. Hence, while the RRM2-4 are necessary for SPEN’s interaction with Xist RNA RepA and its enrichment at the future Xi, IDRs also play a critical role in enhancing its accumulation at the Xi by enabling assembly formations [70,120]. SPEN accumulates very closely to Xist RNA foci, which were found to form at gene-rich regions in the active chromatin [120]. Protein containing accumulations form around the Xi, which were considered supramolecular complexes (SMACs) in a model presented in a recent study [120]. In that study, ~35 Spen and two Xist RNA molecules were counted per SMAC at the Xi, as quantified by the 3D-SIM analysis of mouse epiblast-like cells (EpiLCs) [70,120]. There were in total approximately 50 SMACs per cell, with an estimated ~100 Xist RNA and ~1750 Spen molecules [120]. These numbers for Xist RNA molecules are in agreement with an earlier study, which counted 10 to >100 Xist RNA molecules in the early and late stages of mouse ESC differentiation, respectively [121]. SMACs were found to contain other factors involved in XCI, such as Ciz1, Celf1, Pcgf5, Ezh2, Rybp, and potentially other unidentified proteins [120] (Ciz1 [122,123] and Celf1 [124] assembly formation were also reported in mice previously:). SMACs were shown to constitute a very special environment with a fast exchange but also increased concentrations of their components [120]. XCI-related SMACs might possess liquid–liquid phase separation properties, but to this day, this had not been experimentally demonstrated in cells.

Several interactions have been observed close to the IDRs of SPEN. In the C-terminal part of the long IDR of SPEN is the nuclear receptor interaction domain (RID), where Spen was shown to interact with Msx2 in mice (residues 2070–2394 in mice) (Figure 2) [6]. As mentioned before, Msx1 and Msx2 act as transcriptional repressors in developmental stage-specific gene expression in osteogenesis [6]. Notably, Spen’s involvement in the Msx2 pathway is poorly understood to this day. The RID was shown to be dispensable for SPEN’s function and localization on the Xi upon XCI induction in mouse ESCs [30]. C-terminal to the RID, SPEN interacts with the transcription factor RBP-Jκ in mice and humans [10,71]. RBP-Jκ acts as a switch in transcriptional regulation of the NOTCH signaling pathway. An XCI-related mechanism, however, has not been demonstrated for the RBP-Jκ-binding region.

Considering that SPEN’s IDRs may undergo disorder-to-order transitions or changes in their conformational biases, the properties of this region could change under specific conditions. To gain a comprehensive understanding of this interaction region in the context of its surrounding IDRs, additional studies are required.

## 6. Concluding Remarks and Future Directions

### 6.1. In Vivo and In Vitro Models

Recent extensive research on SPEN has unveiled new functions and mechanisms, highlighting its significance in XCI. We summarize substantial in vitro and in vivo evidence, particularly in mice. This allows us to identify interesting areas for future investigation. When examining SPEN’s function in XCI, there remain gaps in our understanding of its mechanism, and its role in vivo is not yet fully clarified. Two targeted SPEN mutations have been engineered in mice, a truncated SPEN deletion in Kuroda et al. (2003) and a zygotic as well as a conditional oocyte-specific *Spen* knockout in Dossin et al. (2020) [30,71]. The truncated *Spen* deletion suggests a late embryonic lethality phenotype (E12.5), which is later than the embryonic lethality observed in mice with defective imprinted XCI (E8.5). The late lethality could be a consequence of residual truncated proteins, as this was not evaluated, and truncated transcripts were still detectable. Furthermore, the observed defects in the pancreas and heart of *Spen* mutant embryos and the timepoint of embryonic lethality in Kuroda et al. (2003) suggest an impairment of the NCoR/Smrt and/or Notch signaling pathway. Dossin et al. (2020) then demonstrated that zygotic *Spen* knockout impairs imprinted XCI. This observation highlighted Spen’s role in imprinted XCI in addition to its known necessity for random XCI in ESCs [30]. However, none of the two in vivo studies evaluated the embryos’ sex in respect to the lethality or sex-specific phenotypic components that originate from *Spen* knockouts. The investigation of a null mutant embryo could resolve and potentially confirm the time point of embryonic lethality. Furthermore, it could also be compared to the phenotype obtained by the truncating *Spen* mutation of Kuroda et al. (2003) [71]. Differences between the two mutants could resolve some additional questions as to what extent Spen’s mechanism is carried out by the remaining RRMs in the truncating mutants of Kuroda et al. (2003) [71]. Importantly, if *Spen* null mutants were to reveal a late lethality, it could suggest that in vivo, new and redundant factors might be compensating for the loss of Spen.

### 6.2. RNA-Recognition Motifs

When analyzing sub-regions of SPEN, it becomes clear that there are still some aspects that remain open for further investigation. The extension of RRM4 (xRRM4), for example, has not been investigated in regard of SPEN’s mechanism in XCI. The α-helix of many other xRRMs could be demonstrated to have a regulatory or direct involvement in the RNA binding ability and mechanism of the respective protein. Notably, mouse and human RRMs share 93.27–100% sequence identity (Figure 2). This high conservation suggests similar or identical mechanisms of these functional regions in humans and mice. The region with the least, but still conserved, sequence is the second α-helix of xRRM4, which may differ in its function as a result. Therefore, further studies on XIST RNA binding could help elucidate species differences and the precise role of the extending α-helix of SPEN’s xRRM4.

SPEN’s binding to the H12-H13 hairpin substructure of SRA RNA reveals varying dependencies on its different RRMs. RRM1 and RRM2 are likely not involved, whereas RRM3 and RRM4 are both required for RNA interaction [91]. However, it is not clear whether this behavior reflects a general mechanism of SPEN–RNA interaction or if it is a specific mechanism for the interaction with SRA RNA. Results obtained with mRRM3mut suggest that SPEN’s binding to Xist RNA could involve a similar mechanism as its binding to SRA RNA. However, the involvement of RRM2 binding as well as the RRM3/RRM4 co-dependency are still not fully understood in the context of Xist RNA binding.

RRM1 has been excluded from many functional and structural studies. Observations suggest that RRM1 is dispensable for the interactions studied. However, the high conservation of RRM1 implies that it is involved in the function of Spen and is possibly subject to positive evolutionary selection. Consequently, further investigations into the role of RRM1, especially in the context of XCI, would be interesting.

Another open question is which mechanisms or dependencies underlie the specificity of SPEN for XIST RNA RepA. The sequence specificity of SPEN is still a matter of debate. Additionally, XIST RNA RepA exhibits structural features that are likely reoccurring throughout the RNA. Thus, the level of specificity for XIST RNA RepA, as exhibited by SPEN, may not necessarily be expected. Interestingly, RBM15 and RBM15B were demonstrated to not only bind RepA but also other regions of XIST RNA in HEK293 cells [29]. Considering that RBM15 and RBM15B have a highly similar domain topology to SPEN, and their RRM1-3 appear to interact with XIST RNA similar to SPEN’s RRM2-4, this observation highlights functional differences. The difference is likely explained by different amino acid sequences of the RRMs. The mechanisms behind SPEN’s specificity for RepA and simultaneous region-unspecific binding from another SPEN family protein, like RBM15/RBM15B, would be interesting subjects for future investigations.

Furthermore, additional factors might be involved in mediating this specific interaction, such as m6A writers WTAP/METTL3 or RBM15 and RBM15B, which also bind to XIST RNA RepA. Along with these factors, the role of m6A modifications of XIST RNA in regard to its binding abilities and structural consequences are not yet well understood. Additionally, it remains unclear if and how SPEN depends on WTAP-METTL3 recruitment or the presence of RBM15 and/or RBM15B for binding to XIST RNA RepA. A further interesting candidate in respect to the binding of XIST RNA RepA by SPEN is YTHDC1. Since YTHDC1 was shown to interact with XIST RNA, RBM15, RBM15B, and SPEN, there may be a direct or indirect role of YTHDC1 in the SPEN–RepA interaction. At present, a substantial knowledge gap remains regarding the exact mechanism underlying the specific binding of XIST RNA RepA by SPEN’s RRMs. Further research into the dependencies of these processes, as well as the interplay of the m6A machinery and SPEN’s role in XCI in both humans and mice, could contribute to filling this knowledge gap.

### 6.3. SPOC Domain

The SPOC domain of SPEN evidently has a clear role in NCoR/SMRT recruitment, which, in turn, leads to the recruitment of HDAC3 for the subsequent deacetylation of the X chromosome. With an amino acid sequence identity of 99.4% between humans and mice (Figure 2), the SPOC domain comprises a domain with likely highly similar functionality, whereas functional disparities likely stem from differences in interaction partners or differences inherent to the human and mouse systems.

Interestingly, Spen depletion or knockout was demonstrated to lead to a complete abolishment of XCI, while *Hdac3* knockout led to a delayed deacetylation of the Xi in mouse ESCs [24,30]. HDAC3 was suggested to be the only relevant HDAC in XCI [16,24]. Thus, these results suggest a mechanism of SPEN leading to the deacetylation of the Xi, which would be redundant to HDAC3.

Cells with XIST RNA RepA deletion were demonstrated to still form a repressive compartment, and RNA PolII is still excluded from the area [23]. Spen or Hdac3 knockdown in mouse ESCs not only led to a defect in PRC2 recruitment but also a loss of the RNA PolII exclusion from the Xi [16]. These observations indicate an independency of RNA PolII exclusion on XIST RNA RepA. On the other hand, the presence or action of Spen and/or Hdac3 may be relevant for the exclusion of RNA PolII from the repressive compartment. Further research on this process and also on the interaction of SPEN with RNA PolII may help to clarify the mechanisms underlying the RNA PolII exclusion.

### 6.4. Intrinsically Disordered Regions

IDRs have remained the least explored regions of SPEN. Notably, there is no study to this day investigating the IDRs of human SPEN. The disorder score of the IDRs is conserved between human and mouse SPEN by prediction. However, human and mouse amino acid sequences only share 63.26% identity (Figure 2). This needs to be considered when generalizing SPEN IDR function derived from mouse studies to the human mechanism. Nevertheless, recent studies of mouse IDRs revealed that they are important for the proper localization and accumulation of SPEN at the Xi. While these insights are functionally valuable, additional studies are required to investigate not only the functional and regulatory aspects but also the structural role of IDRs.

Interestingly, the phenotype of SPENΔIDR could be rescued to a large extent by fusing another protein’s IDR to the N-terminal end of SPENΔIDR in mouse cells [70]. The replacement of SPEN’s IDRs allowed assembly formation and partial restoration of XCI, as seen in cells with wild-type SPEN. The extent of mechanistic replacement is difficult to estimate with such a rescue experiment since the physicochemical properties of the replacement IDR are not completely clear. However, it opens some additional and interesting questions which require further investigation. For example, it is unclear if the position of the IDR within the protein is relevant to SPEN’s function. The replacement fragment was fused to the N-terminus of SPENΔIDR. Thus, it would be interesting to investigate other locations or a direct replacement of the IDR. Also, it is still not clear if SPEN’s IDRs are targeted to mechanistically relevant PTMs. Although the rescue with a replacement IDR would suggest that SPEN likely does not require specific PTMs, this cannot be excluded due to the unique physicochemical properties of the replacement IDR. PTMs could lead to disorder-to-order transitions of SPEN’s IDR, enabling certain protein–protein interactions. Very speculatively, a regulatory mechanism like this may also offer an explanation for the specific interactions of SPEN with interactors like XIST RNA RepA or could shed light on how SPEN can play specific roles in various cellular pathways. Considering the large size of SPEN, with most of its sequence predicted to be disordered, its IDRs are likely to be highly regulatory and/or functional, which could be an interesting subject of further investigation.

### 6.5. Proposed Model and Open Questions

**Figure 4:**
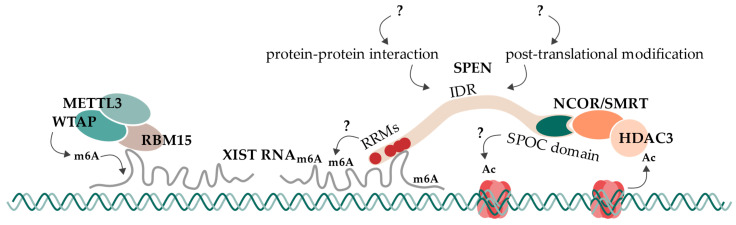
SPEN’s mechanism in the initiation of X chromosome inactivation. SPEN is recruited to the future inactive X chromosome by XIST RNA. It then bridges XIST RNA to the co-receptor complex NCoR/SMRT via interactions on its RNA-recognition motifs (RRMs) and its SPOC domain. NCoR/SMRT interaction activates HDAC3, leading to the deacetylation of nearby histones. This is the first epigenetic change of X chromosome inactivation and is followed by subsequent repressive processes. An additional deacetylation mechanism, which is SPEN-dependent but HDAC3-independent, might exist (indicated with “?”). RBM15 recruits the m6A-writer complex consisting of WTAP and METTL3 to XIST RNA, leading to its subsequent methylation. This methylation likely impacts XIST RNA’s function. However, SPEN’s dependency on m6A modification of XIST RNA or XIST’s interaction with RBM15/WTAP/METTL3 is not clear to this day (indicated with “?”). SPEN’s function might be regulated or extended by further protein–protein interactions or post-translational modifications at its intrinsically disordered regions (IDRs) (indicated with “?”).

Concluding this research summary, present evidence suggests and supports a mechanism of SPEN which bridges the XIST RNA A-repeat to repressive machineries like NCoR/SMRT and HDAC3 (Figure 4). For that, RRM2-4 are involved in A-repeat binding, and the SPOC domain directly interacts with the NCoR/SMRT complex. This observation is supported by several studies in humans and mice. Furthermore, we propose an additional dependency of SPEN for its interaction with XIST RNA A-repeat, which would provide a complete explanation for SPEN’s specificity towards the A-repeat. This could be an additional factor that is required for the interaction (such as WTAP/RBM15/RBM15B/YTHDC1) or factors that enable the interaction by their direct function on XIST RNA (such as m6A modifications). Alternatively, SPEN could be the target of a regulatory PTM, especially in its IDRs. Also, the extending alpha helix of RRM4 could regulate its RNA binding mechanism and/or may also contribute to SPEN’s specificity for the A-repeat. The long IDRs likely enable some functional plasticity of SPEN that has not been considered so far. While the SPOC domain binds the NCoR/SMRT complex, the action via an HDAC3-redundant pathway may be involved in histone deacetylation. This additional pathway may involve the SPOC domain and may also rely on some regulatory function of other regions like IDRs. It will be exciting to see how future research in these areas will further advance our understanding of the role of SPEN in gene regulation and cell signaling.

## Data Availability

Not applicable.
